# Moving academic conferences online: Aids and barriers to delegate participation

**DOI:** 10.1002/ece3.7376

**Published:** 2021-03-16

**Authors:** Cassandra L. Raby, Joah R. Madden

**Affiliations:** ^1^ Association for the Study of Animal Behaviour UK; ^2^ Centre for Research in Animal Behaviour College of Life and Environmental Sciences Washington Singer Labs, University of Exeter Exeter UK

**Keywords:** accessibility, carbon footprint, equality, greenhouse gas emissions, inclusivity, virtual conference

## Abstract

In‐person academic conferences are important to disseminate research and provide networking opportunities. Whether academics attend in‐person conferences is based on the cost, accessibility, and safety of the event. Therefore, in‐person conferences are less accessible to academics and stakeholders that are unable to overcome some of these factors, which then act as a barrier to equal and inclusive participation. Additionally, the carbon footprint of conference travel is increasingly becoming a factor in deciding on whether to attend a conference. Online conferences may provide opportunities to mitigate these challenges. Here, we illustrate how a learned society can move their conference online. Then, comparing data acquired from the virtual conference and previous in‐person conferences, we explore the aids and barriers influencing the decision of delegates to attend the meetings. Ultimately, moving meetings online aids delegate participation by removing concerns about travel, cost, and carbon emissions, but there remains a barrier to participation as online meetings are perceived as less effective for networking and social opportunities.

## Graphical abstract

Whether academics attend in‐person conferences is based on the cost, accessibility, and safety of the event; therefore, in‐person conferences are less accessible to academics and stakeholders that are unable to overcome some of these factors, which then act as a barrier to equal and inclusive participation. Moving meetings online aids delegate participation by removing concerns about travel, cost, and carbon emissions, but there remains a barrier to participation with online meeting being perceived as less effective for networking and social opportunities.

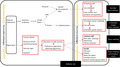

## 1 INTRODUCTION

In‐person academic conferences are important components of a researcher's role through enabling the dissemination of research and providing networking opportunities (de Leon & McQuillin, 2020; Oester et al., 2017). In‐person conferences are the traditional method of conducting scientific meetings, and the decision for attending a conference is primarily based on its cost, accessibility, and safety (Yoo & Chon, 2008; Zhang et al., 2007), and so these factors can act as aids or barriers for academics and stakeholders to attend conferences. The discipline of ecology and evolution is still facing issues surrounding diversity and inclusion, with minoritized groups facing barriers to accessing scientific training and networking opportunities (Cid & Bowser, 2015). In conservation especially, professionals are not proportionally representing the geographical regions that have the greatest biodiversity or greatest need for conservation management, potentially leading to inefficiencies and poor management of the environment (Fraser et al., 2017; Romero & Andrade, 2004). Additionally, this lack of diverse representation at conferences reduces communication, the visibility of minoritized groups, and access to role models that may inspire or mentor early career researchers (Jones et al., 2014; Sardelis et al., 2017). Therefore, calls to remove the barriers that are limiting global networking and communication are vital to improve the inclusivity of ecological and conservation science (Smith et al., 2017).

Steps to make in‐person conferences more inclusive and accessible are seen in organizations such as the British Ecological Society (e.g., through their Equality & Diversity Working Group) and through organizations, including the Association for the Study of Animal Behaviour (ASAB), providing travel grants and childcare for delegates attending their meetings. Contrasting with this, some ecology conferences present barriers to delegate participation because they are held in locations that discriminate against an individual's identity, and the physical accessibility and safety of in‐person conferences are not always considered (Tulloch, 2020). Additionally, traditional conferences can be inaccessible to those from low‐economic countries, those with caring or parental responsibilities, or for delegates with disabilities, perpetuating inequalities that currently persist within academia. In addition to these limitations, the travel to in‐person conferences greatly contributes to an academic's greenhouse gas emissions (Achten et al., 2013; Grémillet, 2008; Spinellis & Louridas, 2013). Researchers, particularly those working in sustainability or conservation, are increasingly deciding whether they should attend a conference based on its contribution to their carbon footprint (Caset et al., 2018; Favaro, 2011; Fraser et al., 2017; Grémillet, 2008; Holden et al., 2017). Overall, in‐person conferences provide barriers for delegate attendance, despite steps to make these events more inclusive, and could therefore be driving inequality within academia.

Online conferences may provide opportunities to mitigate these issues by removing or reducing economic, social, and environmental barriers. Here, we define online or virtual conferences as being any academic meeting that does not occur in the same physical location and instead all delegates meet online. The COVID‐19 pandemic has forced many scientific organizations to move their conferences online (Viglione, 2020), but prior to this research has supported the case that holding meetings on a virtual platform can increase accessibility to academics, for example, to minoritized groups within academia (Black et al., 2020), and as an effective way to decarbonize academia and promote global inclusivity (Fraser et al., 2017). In the wake of COVID‐19, the move of conferences from in‐person to online platforms has provided us with an opportunity to examine the effectiveness and efficiency of virtual conferences in increasing inclusivity when compared to in‐person meetings. To do this, we present a case study of a learned society, Association for the Study of Animal Behaviour (ASAB), comparing the accessibility of their virtual conferences against their in‐person conferences. Specifically, we address whether virtual conferences alter the cost of attending conferences, in terms of both fees and travel; the benefits of networking at conferences; and the environmental impact of conferences.

### 1.1 Case study: conferences hosted by the Association for the Study of Animal Behaviour (ASAB)

The ASAB is a learned society with around 1,500 members, most of whom are academic researchers, but it also includes other teachers, professional animal handlers, and other animal behavior experts. The Society is primarily centered within the UK and Europe (with an independent sister, the Animal Behavior Society in the United States). It hosts three regular meetings annually as well as supporting occasional smaller meetings. These meetings are open to both members and nonmembers with no current discrimination in cost. Two of the traditional in‐person meetings (the Easter and Summer events) have a registration fee between <£100‐£200. However, in‐person Winter Meetings are free and are held at a fixed venue, the Zoological Society of London. These Winter Meetings last for two days, typically attract ~200 delegates, and are open to anyone.

**FIGURE 1:**
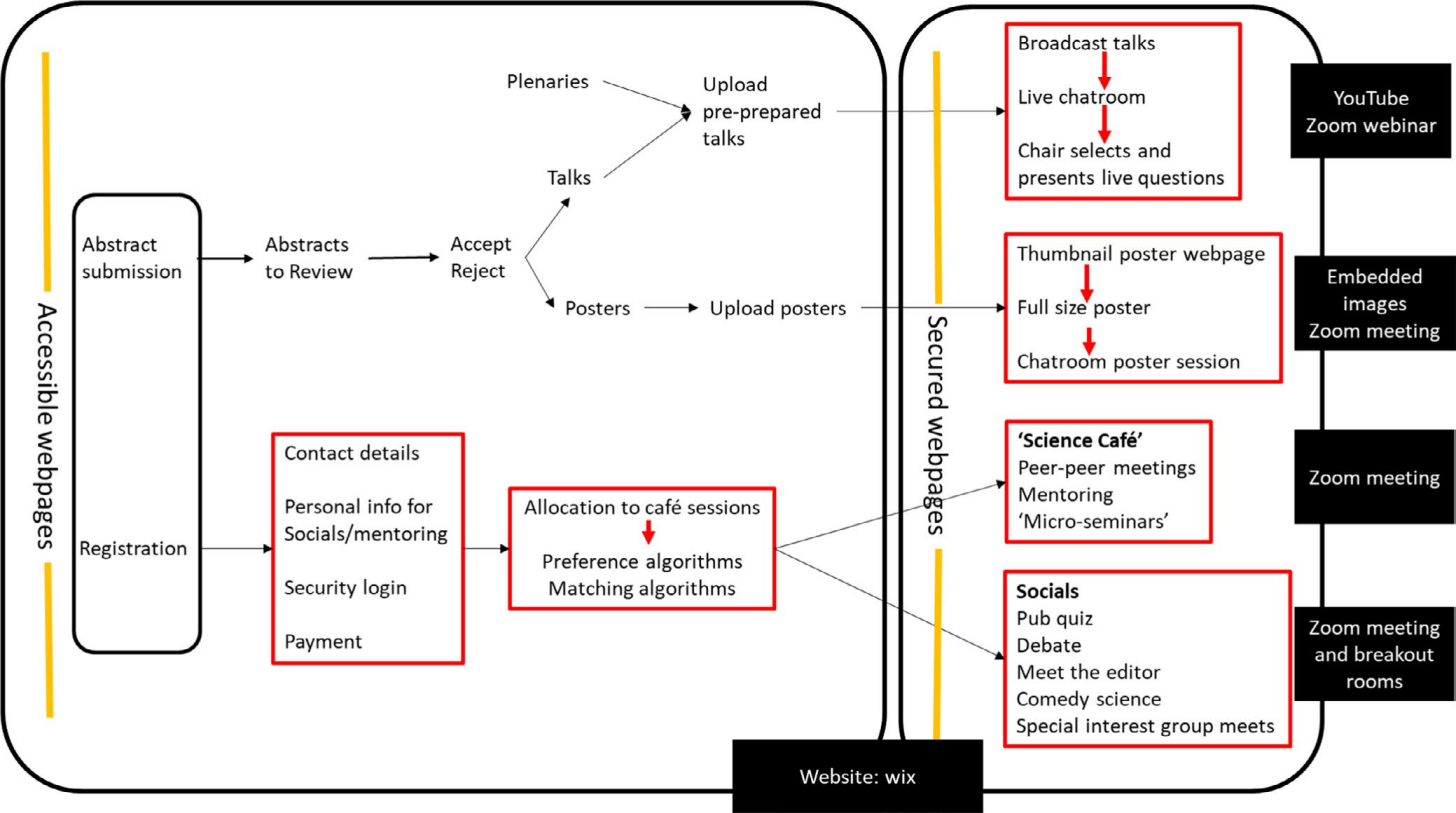
Showing the plans to convert the in‐person conference into an online conference. The text in black boxes shows the technology that we chose in order to achieve these aims

During the COVID‐19 pandemic, ASAB moved their conferences online by developing a conference website where pages with the embedded talks, posters, and links to live sessions were only accessible to registered delegates (Figure 1). The first of these online conferences was held in July 2020 and registration was free and open to both ASAB members and to nonmembers. The conference was themed with plenaries and presentations discussing research associated with: “How do parasites affect behaviour?” The number of registrations and participating delegates from these two meetings showed a large increase in engagement at the online conference (2018 attendance *n* = 204; 2019 attendance *n* = 179; 2020 registration *n* = 950 and attendance *n* = 480; Raby & Madden, 2021). Additionally, when organizing a second ASAB conference online for the Winter 2020 event, there was still an increase in delegate registration and attendance when compared to the in‐person events (registration *n* = 670 and attendance *n* = 400; *unpublished data*) despite conference fees being £15 for nonmembers (and free to ASAB members or delegates from low‐ to middle‐economic countries). Here, we explore the drivers behind the increase in the delegate attendance and explore whether these differences are due to online conferences providing a more inclusive and accessible opportunity when compared to in‐person conferences.

## 2 AIMS

This study compares the accessibility and inclusivity for delegates attending an online ASAB conference (Summer 2020) against a previous ASAB in‐person conference (Winter 2019). We consider the three key areas associated with the decision‐making process of conference attendance: (a) conference costs and registration fees; (b) social costs and opportunities; and (c) the environmental impact.

The aims of the study were to:

- Explore whether the reduced travel costs associated with online conference attendance acts as an aid or barrier to delegates.
- Establish whether the removal of travel opportunities acts as an aid or barrier to delegates.
- Determine whether moving conferences online impacts networking opportunities, and if so, does this act as a barrier to delegates.
- Explore various ways in which flexible attendance influences accessibility.
- Calculate the environmental impact of moving conferences online, and whether delegates are concerned about their carbon footprint when attending in‐person events.

## 3 METHODOLOGY

This study explores how moving conferences online may impact the aims and barriers of delegates in attending academic meetings. To do so, we opportunistically utilized data gathered at the in‐person ASAB winter conference 2019 which were obtained with the primary purpose of informing ASAB’s committee of the carbon footprint of in‐person ASAB events. Delegates arriving at the meeting were asked to report the location they had traveled from and their method of transport, as well as identifying their career stage. For the Summer 2020 meeting held online, we circulated a preconference survey and a postconference survey to registered delegates via email and also on the conference website.

The premeeting questionnaire comprised of a series of forced choices, free choices, Likert scale agreement scores (5‐point scale), and free‐text responses. Similarly, the postmeeting questionnaire comprised of a series of forced choices, free choices, Likert scale agreement scores (7‐point scale), and free‐text responses. We did not offer any inducements to complete the surveys, and all data were anonymized automatically. Additionally, during the meeting, we monitored engagement with the meeting website and delegate geographical location via Wix and Google Analytics. All conference delegates accepted our privacy policy and terms of conditions for the use of cookies on the website and the use of data when registering.

### 3.1 Data analysis: carbon footprint

We estimated the carbon footprint of what ASAB’s online meeting would have been had it been held in real life and all delegates had traveled to London to attend. We used Wix Analytics to identify the countries from which delegates accessed the meeting on the day. Since the online conference is not a true reflection of the in‐person events, we also calculated the carbon footprint of the ASAB’s Winter meeting in 2019. To calculate the travel carbon costs for both of these events, we used https://www.carbonfootprint.com/calculator.aspx to calculate their carbon footprint for road and rail and https://www.carbonindependent.org/22.html to calculate their carbon footprint for air travel.

The online meeting itself likely imposed some carbon costs, in terms of the computing infrastructure necessary to support the meeting (running a website for two months) and connect the 480 delegates and enable them to watch a total of 160 min of YouTube video and participate in 3 hr of Zoom networking. Our engagement data did not permit us to accurately calculate how long each delegate spent engaged in each activity, nor indicate what equipment they were viewing on (mobile/desktop/screen type/viewing resolution), nor their connection method (mobile, Wi‐Fi, or fixed line), all of which are critical determinants of energy consumption (Preist et al., 2019). Therefore, an accurate calculation of the carbon footprint of our meeting is beyond the scope of this paper, but we applied a calculation based on 2011 technologies to determine the carbon outputs of streaming video (Shehabi et al., 2014).

## 4 RESULTS

Here, we provide our findings from in‐person conferences (ASAB’s Winter 2019 conference) when compared with an online conference (ASAB’s Summer 2020 conference) to explore the differences in the aids and barriers that delegates face when deciding to attend these meetings. Firstly, this increase in registrations seen at the online conference occurred across both senior academics and early career and student researchers. The crude composition of the delegates according to their career stage was identical for the in‐person Winter 2019 meeting and the virtual Summer 2020 meeting (based on postsurvey responses) with 21% being tenured (other academic role/senior academic role) and 79% being students, ECRs, or nonacademics.

### 4.1 Survey data

**FIGURE 2:**
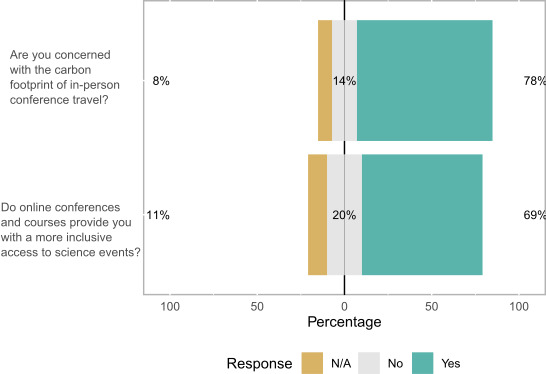
A stacked barplot illustrating the proportion of responses to the preconference questionnaire data

From the preconference survey, we received 179 responses (19% of those registered, *n* = 950). Additionally, for the postconference survey, we received 66 responses (14% of attendees, *n* = 480). The findings from the preconference questionnaire illustrated a strong agreement that online conferences would be more inclusive than in‐person conferences (70% agreed; Figure 2). From the free‐text responses in the surveys, three key elements were cited as being the reason why virtual conferences are more accessible: reduced cost; reduced time traveling; and more flexible approach to accessing the conference content. The following results explore these statements in more detail.

### 4.2 Economic costs and fees

**FIGURE 3:**
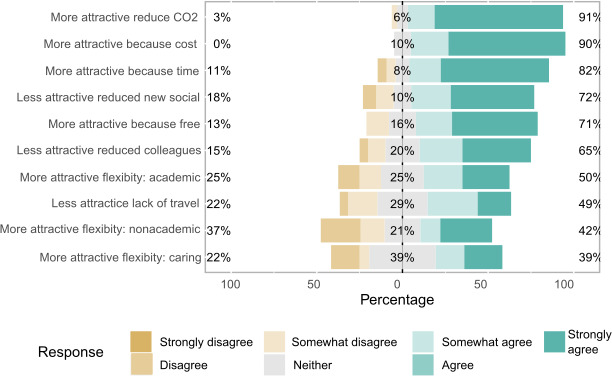
A Likert plot illustrating the proportion of responses to the postconference questionnaire data. The questions asked delegates whether they were more or less likely to attend an online conference depending on particular factors. Full questions are provided in the Appendix

That the meeting was free was important for the delegates. More than two‐thirds of postmeeting survey respondents agreed that the virtual meeting was more attractive than an in‐person meeting because it was free (69% agreed; Figure 3). A similar number (73%) thought that future ASAB meetings should also be free. However, when we asked what an appropriate fee would be for such a day, just 13% replied that they would be willing to pay only £0. We interpret this as meaning that if there had been a charge, they would not have attended. The other 87% considered it appropriate to pay a charge to participate. These included 34% suggesting a fee of £1–10, 39% suggesting a fee of £11–25, and 14% suggesting a fee of £26–50. Several survey respondents remarked that costs should be differential based on career stage, location, or contract permanency. Others suggested that funding could be via voluntary donations.

Benefits or justifications for providing a free meeting reported by respondents fell into two main areas. First, some delegates stated that a free meeting increased accessibility and diversity with statements such as: “Keeping meetings free makes it much more accessible to students and early career researchers from third world countries (like India),” “Not charging makes it more accessible, especially for researchers/students based in developing countries,” “Charging for meeting attendance creates an exclusionary and elitist environment, and small costs should be covered by membership fees.” Second, some delegates stated that because online meetings incurred low or negligible running costs, charging could not be justified with statements such as: “Conference organisers are not usually paid for their work, so what would the fee go towards? No room bookings, food, socials to provide...”; “If the conference is in person then I expect to pay but if it is online I expect it to be free.” A third benefit of not charging that a couple of delegates mentioned was that it encouraged researchers from outside the field to participate on a speculative basis: “The topic of the meeting is not my area, and I only joined the meeting because it was free. I'm really glad I did because not only did I learn loads about something I knew very little about.”

### 4.3 Flexibility and travel opportunities

The ability for delegates to attend without leaving their homes or workplaces was generally reported to be attractive by respondents. Respondents agreed that virtual meetings were more attractive than real‐life ones because they permitted flexible participation during the day, freeing the respondent for other academic work (58% agreed; Figure 3). However, this flexibility was not considered to be so important when considering facilitating nonacademic work (45% agreed; Figure 3), or caring duties (31% agreed; Figure 3). It should be noted that many delegates may have no nonacademic work to perform or caring duties to commit to at that time. These benefits were reflected in statements by respondents such as “The flexibility this conference offered was amazing. I did not have to pre‐plan travel and accommodation well in advance, spend half a day traveling and arrive exhausted, and then have to sit still for X time to listen to the talks, which not only saved me money, but so much time and energy as well.” For some respondents, not visiting a new venue was seen as an adverse consequence of the virtual format, and 52% agreed (Figure 3) that the virtual meeting was less attractive because of the lack of justification to visit another university/town/country. In contrast, the reduced need to travel reducing economic costs was agreed to make the virtual meeting more attractive by a greater amount (86% agreed; Figure 3), while slightly fewer agreed that the virtual meeting was more attractive because reduced travel reduced time costs (77% agreed; Figure 3).

### 4.4 Social costs and opportunities

The major social cost that we identified was the reduction in personal connections, whether professional or informal. Postmeeting survey respondents overwhelmingly agreed that virtual meetings were less attractive because it was harder to interact socially with established colleagues (75% agreed; Figure 3) or because it was harder to meet new colleagues (74% agreed; Figure 3). This lost opportunity to socialize was reflected in the respondent's statements: “I really found the almost complete lack of social contact a bit soul destroying.”; “Sadly, I personally experienced that I was veeeery [sic] much less engaged in this online meeting compared to a real‐life meeting. I cannot really say why but I think this was due to sitting at home alone and not ‘feeling the conference spirit’, so to say.” However, these feeling were not ubiquitous, with at least some respondents that engaged in planned social events (Science Café, Quizzes) reporting that they found socializing online easier than in real life: “In a live conference, it is often ‘weird’ to go up to random people and network with them—being put together in a zoom meeting room is better at breaking the ice and initiating conversation.” “It was actually easier to meet new people (I joined the science cafe) then perhaps in an in‐person conference.” This improved socializing appears to have been facilitated by targeted and selective groupings of delegates in some of the social and professional development sessions, with respondents reporting: “Assigning groups according to research interests (science cafe) was really helpful”; “The [participants] of the Science cafe‐group were well selected according to main research interests. And thus, exchanging research and socializing was very useful.” The use of the quiz at the end of the day also provided an opportunity to establish new friendships: “[The] quiz was great way to socialize, have fun and meet new people.”; “[the] pub quiz was just fun and a nice way to end the conference on a high.”

### 4.5 Carbon footprint

Of the 417 delegates for which we had location data (87% of those that attended, *n* = 480), 35 countries were represented, with 303 (73%) delegates from Europe (including 188 from UK—45%), 54 (13%) from N America, 28 (7%) from Asia, 19 (5%) from S America, 10 (2%) from Australasia, and 3 (<1%) from Africa. We crudely calculated the carbon footprint that these delegates would have imposed had they traveled to the meeting if held in London, UK. We assumed that each delegate would have flown directly from their nations’ capital to London. This suggested that, in total, delegates would have traveled at least 2,040,736km producing at least 234.7 tonnes of CO_2_. Given that the online meeting attracted many international delegates which was unusual for an ASAB meeting and may not reflect actual patterns of attendance at equivalent face‐to‐face meetings, we also compared the online carbon footprint with that of the most recent equivalent free real‐life meeting. We collected data from 172 delegates (129 UK—75%, 38 EU—22%, 5 International—3%). Twenty delegates lived in London so were deemed to impose no carbon costs. A total of 102 delegates traveled 59,072km by train producing 2.19 tonnes CO_2_. Seven delegates traveled 3,744 km by bus, coach, or car producing 0.39 tonnes CO_2_. Forty‐three delegates traveled 143,744 km by plane producing 16.53 tonnes CO_2_. Therefore, the total distance traveled by all delegates was 206,560 km and produced 19.11 tonnes CO_2_.

Comparatively, we calculated the carbon footprint of the online conference based on 2011 technologies suggesting a cost of streaming video of 0.42 kgCO_2_/hour (likely now far more efficient) (Shehabi et al., 2014), assuming that all delegates engaged in all online activities. We might presume that a high estimate of the meeting's carbon footprint was 1.1 tonnes CO_2_. Therefore, the online meeting produced less than 0.4% of what the same meeting held in real life would have produced and less than 6% of the carbon footprint of one of our previous in‐person meetings.

### 4.6 Environmental costs

The benefits of a reduced carbon footprint were agreed by delegates to be important in making a virtual meeting more attractive than a real‐life one. In the preconference survey, 78% of delegates (Figure 2) felt concerned about the carbon footprint of in‐person conferences and over 85% of postmeeting respondents agreed (Figure 3) that a virtual meeting was more attractive than a real‐life meeting because it removed the need to travel, specifically reducing carbon footprint. No respondents in the postmeeting survey mentioned the carbon footprint in their free‐text responses, but four of the 179 premeeting survey respondents commented including: “We are working with nature, we should give the example by trying to reduce our carbon footprint. This method is great for that.”; “Even if different from in‐person conferences, I strongly support virtual conferences for easier access to science and lower carbon footprint”; “I work in a conservation field, and I am quite concerned about my carbon footprint. I appreciate an opportunity to engage with others without adding to that global burden.”; “I think ASAB should make EVERY conference virtual. We cannot expect others to take climate change seriously if we don´t practice what we preach.”

## 5 DISCUSSION

The delivery of an online Society scientific meeting proved to be more economical, more environmentally friendly, and more inclusive when compared with a recent real‐life free society meeting. The number of delegates participating was almost 300% higher than expected at a comparable meeting. International representation was wider with delegates attending from 35 countries compared with 15 and with 45% of virtual delegates from the UK as opposed to 75% at the in‐person meeting. The virtual format did not appear to alter the delegate composition according to career stage. It imposed no travel or accommodation costs on delegates. The carbon footprint of the meeting was less than 1% of what would have been incurred if the same event had occurred in‐person. Being online allowed delegates to participate from home while still engaged in other academic work or caring duties, or even engage with the conference effectively without being exhausted by traveling. Delegates expect future conferences to be free, and below, we discuss the overall cost comparison of ASAB meetings. Overall, these benefits were achieved with little perceived reduction in the scientific content of the meeting and were widely recognized by delegates attending the meeting. The main barrier of the conference was the ability of delegates to socialize with each other, and specific details of how to improve this are available in Raby & Madden (2021).

### 5.1 Economic costs and fees

One striking issue that may have come about from providing the online conference without any fees was the high number of registrations, but a comparatively lower number of delegates actually engaging with the conference. Of the 950 delegates that registered to attend the conference, around half were actually “present” on website on the day of the conference (Raby & Madden, 2021). This provided us with some financial challenges. The cost of providing the webinars in Zoom depends on the number of attendees, and while we paid for a capacity of 1,000 people, the number of attendees to the live Q&A sessions was significantly lower than expected (Raby & Madden 2021). Hosting a conference without any registration fees might reduce the opportunities and flexibility of events that can be provided, whereas introducing just a small fee could help cover such uncertainty and recuperate those costs. Furthermore, a small cost could help instill a commitment to participate for the delegate. However, delegates from low‐income countries and/or with small funding budgets felt that the free online conference gave them an opportunity to engage with a conference that they would have otherwise have not attended, so careful attention should be made to ensure that any online conferences with registration fees also try to support open access to delegates with limited funding.

Moving conferences online provide the opportunity for scientific groups to reduce their expenditure and remove many of the obstacles associated with organizing a scientific meeting (e.g., deciding on an accessible, attractive, and environmentally friendly location; Parsons, 2015). ASAB never aims to make financial profit from these meetings (its income is based on membership subscriptions, journal (Animal Behaviour) revenue, and investments). It offers members attending ASAB meetings Conference Attendance Grants (up to £500) that cover travel and accommodation and Childcare Grants (up to £500). It also covers costs of plenary speakers and may contribute to other meeting costs. For the Winter Meeting, it covers costs of venue hire and social events. Some costs (typically < £1000/meeting) may be offset by sponsorship by publishers or manufacturers of equipment or technology of interest to animal behavior researchers in exchange for space where they can sell their wares or demonstrate their equipment.

Comparatively, we decided to develop our own platform to host the virtual meeting, our costs for running the meeting were webhosting (Wix plan: £115, including URL domain) and one month licenses for Zoom webinar access (≤1,000 people = £330). These costs, or similar, will be borne for every subsequent virtual meeting. We also incurred the cost of employing someone to develop and run the website. A technology developer was employed for two months on a PDRA salary level contract (~£3,680 per month). This cost can be seen as an investment and thus spread over forthcoming meetings. Therefore, if the platform that we built serves a further three meetings, then the mean cost per meeting will be ~£2,200. This figure falls below the usual cost to ASAB for the comparable free Winter Meetings of venue hire (~£15,000), plenary travel and accommodation (~£5,800), and member travel and childcare grants (~£1,200). Therefore, the single virtual meeting cost 30% of the in‐person meeting and this cost is expected to drop to 8% as the virtual platform is reused. Consequently, for ASAB providing virtual meetings for free appears to be economically sustainable in at least the medium term. If we had acted independently of ASAB and had to cover our costs, then the total costs (sharing the development costs over four such meetings) spread between all those that registered would indicate a delegate cost of £3.60 or if spread between those that actually attended, a delegate cost of £7.50. If we had decided to impose a charge, then a delegate fee of £10, acceptable to 87% of respondents, could have grossed us £9,500 from those that registered or £4,800 from those that participated. A delegate fee of £25 acceptable to 53% of respondents could have grossed us £23,750 from those that registered or £12,000 from those that participated. However, historically virtual conferences have generally not charged (Anderson, 1996), and an understanding of how much delegates are willing to pay helps guide conferences that are considering financing the conference through registration fees. If necessary, an organization costs for hosting a conference could be £0, but that would require removing the interactive aspects of the conference and reducing the aesthetics of the hosting website (i.e., no domain name, allow the website host to keep adverts on the website), or conferences could be hosted by a third party website developer with costs quoted as being between £8,000 and £20,000.

### 5.2 Flexibility and travel opportunities

While many academics may enjoy the ability and privilege to travel somewhere new, this is only the case if the conference is in a location that is not too costly, time‐consuming, or dangerous to travel to (Yoo & Chon, 2008). As we have illustrated here, the flexibility of the online conference was important to the delegates at our meeting and a key motivator for their attendance. However, our survey may be biased toward delegates that benefited from either flexibility, ease, or inclusivity of online meetings. We may have been unable to capture the opinions of academics who are motivated and unrestricted to visit in‐person conferences. Yet, we can assume that online conferences are more attractive to academics as we saw a huge increase in the number of delegates participating in the meeting when compared to other free in‐person ASAB conferences. This has also been seen at other conferences, (e.g., European Geosciences Union saw an increase from 16,000 to 26,000 delegates; Klöwer et al., 2020). If online conferences continue to see a higher number of registrations than their in‐person equivalents, this may illustrate that online conferences provide fewer barriers for conference attendance.

### 5.3 Social costs and opportunities

Visibility and networking at conferences is considered a key motivator for attendees of in‐person events and a lot of value is placed on conferences providing this opportunity (Oester et al., 2017; Rowe, 2018; Yoo & Chon, 2008). Yet the ability of our online conference to enable social connections was met with mixed opinions. Some delegates felt that the organized structure of the social events meant that they were more willing to participate, whereas others felt this to be inflexible and restrictive, and therefore unable to replicate the same networking opportunities as in‐person events. Communication failures between delegates also occurred and were summed up by one respondent: “What is quite missing is the interaction between the many non‐presenting delegates. To my mind, probably about 90% of all communication that occurs at a conference, including initiation of collaborations, casual discussions and generation of research ideas occurs outside of the presenter‐viewer interaction. That is hard to replace.” This was a recurrent theme and provoked the strongest negative responses to the virtual format. Developing ways to engage delegates and facilitate networking is clearly essential and we tried a range of approaches (science cafes connecting researchers of similar interests, mentoring groups connecting researchers at similar career stages, a continuous Twitter commentary and engagement throughout the meeting, and a quiz at the end of the meeting, see Figure 1). Unfortunately, these apparently failed to capture the spontaneous and serendipitous encounters found between seated neighbors, or in the hallways of conference centers. Despite concerns that online conferences are unable to replicate social opportunities for those that can attend in‐person conferences (also see Oester et al., 2017), it greatly increases the opportunities for researchers who would otherwise have been unable to travel or afford the event. The larger number of attendees, the increase in international delegates, and the structured format of top‐down organized events at our online meeting enabled a more inclusive social environment that captured a much wider academic network.

### 5.4 Carbon footprints

Unsurprisingly, the carbon footprint of the online conference was significantly lower than in‐person meetings. If the exact same conference had occurred in‐person then the carbon footprint would have been an estimated 200 times greater than the online conference. Since it is unlikely that delegates would travel so far for the in‐person conference, we were able to compare the carbon footprint to a previous in‐person ASAB conference, also without registration fees. Here, the virtual conference was 17 times (6%) lower in its carbon output than the in‐person conference. This may be an underestimate compared to other conferences, where food and accommodation should also be accounted for (Bossdorf et al., 2010). However, our estimates of the reduction in carbon footprint are similar than previous estimations of virtual conferences being 7% the CO_2_ costs as in‐person conferences (Ong et al., 2014). The removal of intercontinental flights would be a key factor in reducing carbon emissions for in‐person conferences, and yet it comes at the cost of building an international network for collaboration. However, the opportunities for international networking are not available to all academics, biasing opportunities for academics from high‐income countries. Additionally, our significant reduction in greenhouse gas emissions appears to have provided an aid for supporting academic attendance to conferences, with more delegates registering than have done at previous in‐person conferences. Virtual conferences are a demonstrably effective way of reducing the environmental costs of academic meetings; however, it should be noted that even in‐person conferences could attempt to reduce their carbon footprint in the future (see (Abbott, 2020; Bossdorf et al., 2010; Klöwer et al., 2020; Stroud & Feeley, 2015).

### 5.5 Environmental costs

Many of the conference delegates stated that they were concerned with their carbon footprint when attending in‐person conferences and that the reduced outputs were an advantage to the online conference format. In previous ASAB material (Society newsletter, discussions at AGM, and presentations at previous meetings), we had made delegates aware of the carbon cost of in‐person meetings. We also had a brief statement relating to carbon footprints on the conference homepage (“After the cancellation of conferences and events due to COVID‐19, and mindful of our responsibilities to reduce our carbon footprint, we are trialing this new format”). Therefore, delegates may have been primed in their answering. That said, the call to researchers, and particularly conservation biologists, to reduce their international travel to academic meetings is not new (e.g., Bossdorf et al., 2010; Caset et al., 2018; Fox et al., 2009; Green, 2008; Grémillet, 2008; Klöwer et al., 2020; Rockwell et al. 2020; Stroud & Feeley, 2015). The discussion of moving conferences online has been circulating for some time, but for many learned societies, it has been a global pandemic that has caused a surge in the adaptation to these new events.

### 5.6 Moving forward

“The virtual conference will be different, yes, but not necessarily in a disadvantaging way. I would not have been able to attend in person, thus the virtual concept gains a lot of value. Aiming for a balance between virtual and in‐person conferences might be the best way forward!”

Here, we have provided strong evidence that online conferences can reduce barriers and aid an inclusive, environmentally friendly, and cheaper alternative to in‐person conferences. Their limitations are primarily due to the inability to replicate networking and social opportunities, but this is compensated for with the event being larger and more international. Hosting a conference online is much cheaper for both hosting organization and delegates themselves. As academics and other stakeholders working within a publicly funded sector, we should take these opportunities to greatly reduce our expenditure and aim to redesign conferences to be a “sustainable educational activity” (Rowe, 2019). In fact, the free cost of the conference was a great motivation for many of the delegates and helped create an event that was better attended and more international than previous comparable, free in‐person ASAB conferences. Furthermore, the environmental impact of this event was significantly smaller than that of the equivalent, or previous, in‐person conferences. Although online conferences reduce the opportunities to travel to new cities and academic institutions, many of the conference delegates (78%–85%) felt that online conferences were more attractive than in‐person conferences because of their reduced carbon footprint. In conclusion, moving meetings online aids delegate participation by removing concerns about travel, cost, and carbon emissions, but there remains a barrier to participation with online meeting being perceived as less effective for networking and social opportunities.

## CONFLICT OF INTEREST

All authors state that there is no conflict of interest.

## AUTHOR CONTRIBUTIONS

Cassandra L. Raby: conceptualization (equal); data curation (equal); methodology (equal); writing (equal). Joah R. Madden: conceptualization (equal); data curation (equal); methodology (equal); writing (equal).

## Data Availability

All data have been archived and made available at https://doi.org/10.5061/dryad.cnp5hqc48

## APPENDIX 1. The heading of the question as it appears in , alongside the full questions that were included in the postconference questionnaire.

“More attractive reduce CO2”: The virtual meeting was MORE attractive to me than a 'real‐life' meeting because: Removed need to travel / reduced carbon footprint

“More attractive because cost”: The virtual meeting was MORE attractive to me than a 'real‐life' meeting because: Removed need to travel and reduced economic costs

“More attractive because time”: The virtual meeting was MORE attractive to me than a 'real‐life' meeting because: Removed need to travel and reduced time costs

“Less attractive reduced new social”: The virtual meeting was LESS attractive to me than a 'real‐life' meeting because: Harder to meet new colleagues

“More attractive because free”: The virtual meeting was MORE attractive to me than a 'real‐life' meeting because: it was free

“Less attractive reduced colleagues”: The virtual meeting was LESS attractive to me than a 'real‐life' meeting because: Harder to interact socially with established colleagues

“More attractive flexibility: academic”: The virtual meeting was MORE attractive to me than a 'real‐life' meeting because: it permitted flexible participation during the day freeing me for other academic work

“Less attractive lack of travel”: The virtual meeting was LESS attractive to me than a 'real‐life' meeting because: Lack of justification to visit another University/town/country as part of the conference trip

“More attractive flexibility: nonacademic”: The virtual meeting was MORE attractive to me than a 'real‐life' meeting because: it permitted flexible participation during the day freeing me for other nonacademic work

“More attractive flexibility: caring”: The virtual meeting was MORE attractive to me than a 'real‐life' meeting because: it permitted flexible participation during the day freeing me for caring duties

## References

[ece37376-bib-0001] Abbott, A. (2020). Low‐carbon, virtual science conference tries to recreate social buzz. Nature, 577(7788), 13. 10.1038/d41586-019-03899-1 31871326

[ece37376-bib-0002] Achten, W. M. J. , Almeida, J. , & Muys, B. (2013). Carbon footprint of science: More than flying. Ecological Indicators, 34, 352–355. 10.1016/j.ecolind.2013.05.025

[ece37376-bib-0003] Anderson, T. (1996). The virtual conference: Extending professional education in cyberspace. International Journal of Educational Telecommunications, 2, 121–135.

[ece37376-bib-0004] Black, A. L. , Crimmins, G. , Dwyer, R. , & Lister, V. (2020). Engendering belonging: Thoughtful gatherings with/in online and virtual spaces. Gender and Education, 32(1), 115–129. 10.1080/09540253.2019.1680808

[ece37376-bib-0005] Bossdorf, O. , Parepa, M. , & Fischer, M. (2010). Climate‐neutral ecology conferences: Just do it! Trends in Ecology and Evolution, 25(2), 61. 10.1016/j.tree.2009.09.006 19818525

[ece37376-bib-0006] Caset, F. , Boussauw, K. , & Storme, T. (2018). Meet & fly: Sustainable transport academics and the elephant in the room. Journal of Transport Geography, 70, 64–67.

[ece37376-bib-0007] Cid, C. R. & Bowser, G. (2015). Breaking down the barriers to diversity in ecology. Frontiers in Ecology and the Environment, 13(4), 179. 10.1890/1540-9295-13.4.179

[ece37376-bib-0008] de Leon, F. L. L. & McQuillin, B. (2020). The Role of conferences on the pathway to academic impact. Journal of Human Resources, 55(1), 164–193. 10.3368/jhr.55.1.1116-8387R

[ece37376-bib-0009] Favaro, B. (2011). A carbon code of conduct for science. Science, 344(6191), 1461. 10.1126/science.344.6191.1461 24970073

[ece37376-bib-0010] Fox, H. E. , Kareiva, P. , Silliman, B. , Hitt, J. , Lytle, D. A. , Halpern, B. S. , Hawkes, C. V. , Lawler, J. , Neel, M. , Olden, J. D. , Schlaepfer, M. A. , Smith, K. , Tallis, H. (2009). Why do we fly? Ecologists’ sins of emission. Frontiers in Ecology and the Environment, 7(6), 294–296. 10.1890/09.WB.019

[ece37376-bib-0011] Fraser, H. , Soanes, K. , Jones, S. A. , Jones, C. S. , & Malishev, M. (2017). The value of virtual conferencing for ecology and conservation. Conservation Biology, 31(3), 540–546. 10.1111/cobi.12837 27624673

[ece37376-bib-0012] Green, M. (2008). Are international medical conferences an outdated luxury the planet can’t afford? Yes. BMJ, 336(7659), 1466. 10.1136/bmj.a358 18583676 PMC2440857

[ece37376-bib-0013] Grémillet, D. (2008). Paradox of flying to meetings to protect the environment. Nature, 455(7217), 1175. 10.1038/4551175a 18971997

[ece37376-bib-0014] Holden, M. H. , Butt, N. , Chauvenet, A. , Plein, M. , Stringer, M. , & Chadès, I. (2017). Academic conferences urgently need environmental policies. Nature Ecology and Evolution, 1(9), 1211–1212. 10.1038/s41559-017-0296-2 29046545

[ece37376-bib-0015] Jones, T. M. , Fanson, K. V. , Lanfear, R. , Symonds, M. R. E. , & Higgie, M. (2014). Gender differences in conference presentations: A consequence of self‐selection? PeerJ, 1, 1–15. 10.7717/peerj.627 PMC4207199 25346879

[ece37376-bib-0016] Klöwer, M. , Hopkins, D. , Allen, M. , & Higham, J. (2020). An analysis of ways to decarbonize conference travel after COVID‐19. Nature, 583(7816), 356–359. 32669689 10.1038/d41586-020-02057-2

[ece37376-bib-0017] Oester, S. , Cigliano, J. A. , Hind‐Ozan, E. J. , & Parsons, E. C. M. (2017). Why conferences matter—an illustration from the international marine conservation congress. Frontiers in Marine Science, 4(AUG), 1–6. 10.3389/fmars.2017.00257

[ece37376-bib-0018] Ong, D. , Moors, T. , & Sivaraman, V. (2014). Comparison of the energy, carbon and time costs of videoconferencing and in‐person meetings. Computer Communications, 50, 86–94. 10.1016/j.comcom.2014.02.009

[ece37376-bib-0019] Parsons, E. C. M. (2015). So you think you want to run an environmental conservation meeting? Advice on the slings and arrows of outrageous fortune that accompany academic conference planning. Journal of Environmental Studies and Sciences, 5(4), 735–744. 10.1007/s13412-015-0327-8

[ece37376-bib-0020] Preist, C. , Schien, D. , & Shabajee, P. (2019). Evaluating sustainable interaction design of digital services: The case of YouTube. Conference on Human Factors in Computing Systems ‐ Proceedings, 1–12.

[ece37376-bib-0021] Raby, C. L. & Madden, J. R. (2021). Moving academic conferences online : Understanding patterns of delegate engagement. Ecology and Evolution, (July 2020), 1–9. 10.1002/ece3.7251 PMC8057334 33898013

[ece37376-bib-0022] Rockwell, G. , Rossier, O. , & Miya, C. (2020). ‘Greening’ academic gatherings: A case for econferences. Right Research: Modelling Sustainable Research Practices in the Anthropocene, (October 2019). 10.11647/OBP.0213

[ece37376-bib-0023] Romero, C. & Andrade, G. I. (2004). International conservation organizations and the fate of local tropical forest conservation initiatives. Conservation Biology, 18(2), 578–580. 10.1111/j.1523-1739.2004.00397.x

[ece37376-bib-0024] Rowe, N. (2018). ‘When you get what you want, but not what you need’: The motivations, affordances and shortcomings of attending academic/scientific conferences. International Journal of Research in Education and Science, 4(2), 714–729. 10.21890/ijres.438394

[ece37376-bib-0025] Rowe, N. E. (2019). The economic cost of attending educational conferences. International Journal on Social and Education Science, 1(1), 30–42.

[ece37376-bib-0026] Sardelis, S. , Oester, S. , & Liboiron, M. (2017). Ten strategies to reduce gender inequality at scientific conferences. Frontiers in Marine Science, 4(JUL), 1–6. 10.3389/fmars.2017.00231

[ece37376-bib-0027] Shehabi, A. , Walker, B. , & Masanet, E. (2014). The energy and greenhouse‐gas implications of internet video streaming in the United States. Environmental Research Letters, 9(5), 54007. 10.1088/1748-9326/9/5/054007

[ece37376-bib-0028] Smith, N. S. , Côté, I. M. , Martinez‐Estevez, L. , Hind‐Ozan, E. J. , Quiros, A. L. , Johnson, N. , Green, S. J. , Cornick, L. , Shiffman, D. , Malpica‐Cruz, L. , Gleason Besch, A. , Shiel‐Rolle, N. (2017). Diversity and inclusion in conservation: A proposal for a marine diversity network. Frontiers in Marine Science, 4(AUG), 1–7. 10.3389/fmars.2017.00234

[ece37376-bib-0029] Spinellis, D. & Louridas, P. (2013). The carbon footprint of conference papers. PLoS One, 8(6), 6–13. 10.1371/journal.pone.0066508 PMC3694072 23840496

[ece37376-bib-0030] Stroud, J. T. & Feeley, K. J. (2015). Responsible academia: Optimizing conference locations to minimize greenhouse gas emissions. Ecography, 38(4), 402–404. 10.1111/ecog.01366

[ece37376-bib-0031] Tulloch, A. I. T. (2020). Improving sex and gender identity equity and inclusion at conservation and ecology conferences. Nature Ecology and Evolution, 4(10), 1311–1320. 10.1038/s41559-020-1255-x 32747775

[ece37376-bib-0032] Viglione, G. (2020). A year without conferences? How the coronavirus pandemic could change research. Nature, 579(7799), 327–328. 32184489 10.1038/d41586-020-00786-y

[ece37376-bib-0033] Yoo, J.‐ J.‐E. & Chon, K. (2008). Factors affecting convention participation decision‐making: Developing a measurement scale. Journal of Travel Research, 47(1), 113–122. 10.1177/0047287507312421

[ece37376-bib-0034] Zhang, H. Q. , Leung, V. , & Qu, H. (2007). A refined model of factors affecting convention participation decision‐making. Tourism Management, 28(4), 1123–1127. 10.1016/j.tourman.2006.07.008

